# Biodegradable polymeric insulin microneedles – a design and materials perspective review

**DOI:** 10.1080/10717544.2023.2296350

**Published:** 2023-12-26

**Authors:** Melbha Starlin Chellathurai, Syed Mahmood, Zarif Mohamed Sofian, Cheng Wan Hee, Ramkanth Sundarapandian, Haja Nazeer Ahamed, C. S. Kandasamy, Ayah R. Hilles, Najihah Mohd Hashim, Ashok Kumar Janakiraman

**Affiliations:** a Department of Pharmaceutical Technology, Faculty of Pharmacy, Universiti Malaya, Kuala Lumpur, Malaysia; b Centre for Natural Products Research and Drug Discovery (CENAR), Universiti Malaya, Kuala Lumpur, Malaysia; c Faculty of Health and Life Sciences, INTI International University, Nilai, Malaysia; d Department of Pharmaceutics, Karpagam College of Pharmacy, Coimbatore, India; e Crescent School of Pharmacy, B.S. Abdur Rahman Crescent Institute of Science and Technology, Vandalur, Chennai, India; f Department of Pharmacognosy, Karpagam College of Pharmacy, Coimbatore, India; g INHART, International Islamic University, Kuala Lumpur, Malaysia; h Department of Pharmaceutical Chemistry, Faculty of Pharmacy, Universiti Malaya, Kuala Lumpur, Malaysia; i Department of Pharmaceutical Technology, Faculty of Pharmaceutical Sciences, UCSI University, Kuala Lumpur, Malaysia

**Keywords:** Diabetes, transdermal insulin delivery, oral insulin microneedles, biopolymeric insulin microneedles, implantable insulin microneedles, non-parenteral insulin delivery

## Abstract

Microneedle (MN) delivery devices are more accepted by people than regular traditional needle injections (e.g. vaccination) due to their simplicity and adaptability. Thus, patients of chronic diseases like diabetes look for alternative pain-free treatment regimens circumventing regular subcutaneous injections. Insulin microneedles (INS-MNs) are a thoughtfully researched topic (1) to overcome needle phobia in patients, (2) for controlled delivery of the peptide, (3) decreasing the frequency of drug administration, (4) to ease the drug administration procedure, and (5) thus increasing patient adherence to the treatment dosage regimes. MNs physically disrupt the hard outer skin layer to create minuscule pores for insulin (INS) to pass through the dermal capillaries into the systemic circulation. Biodegradable polymeric MNs are of greater significance for INS and vaccine delivery than silicon, metal, glass, or non-biodegradable polymeric MNs due to their ease of fabrication, mass production, cost-effectiveness, and bioerodability. In recent years, INS-MNs have been researched to deliver INS through the transdermal implants, buccal mucosa, stomach wall, intestinal mucosal layers, and colonic mucosa apart from the usual transdermal delivery. This review focuses on the design characteristics and the applications of biodegradable/dissolvable polymeric INS-MNs in transdermal, intra-oral, gastrointestinal (GI), and implantable delivery. The prospective approaches to formulate safe, controlled-release INS-MNs were highlighted. Biodegradable/dissolvable polymers, their significance, their impact on MN morphology, and INS release characteristics were outlined. The developments in biodegradable polymeric INS-MN technology were briefly discussed. Bio-erodible polymer selection, MN fabrication and evaluation factors, and other design aspects were elaborated.

## Graphical Abstract

**Figure:**
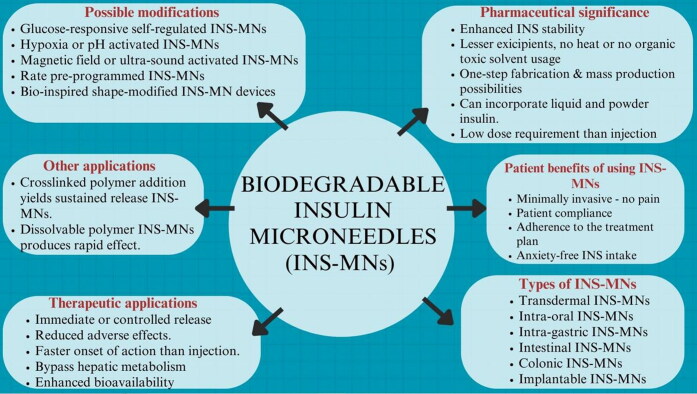

## 1. Introduction

Diabetes mellitus is a complex chronic glucose metabolic disorder characterized by disproportionately elevated blood glucose levels (BGLs). This is either due to the destruction of pancreatic β-cells (in the Islets of Langerhans), producing insufficient insulin (INS) (type 1 diabetes – T1D), or because of INS resistance leading to ineffective INS response (type 2 diabetes – T2D). Diabetes results in an increased risk of complications like immediate diabetic ketoacidosis (DKA), to long-term macrovascular effects like cardio-, cerebro-, and peripheral vascular diseases. Long-term microvascular effects include retinopathy, nephropathy, neuropathy, etc. (Jin et al., 2018). Exogenous INS is the widely accepted standard anti-diabetic treatment option given as subcutaneous (S.C.) injections. Patient compliance to injections is lower due to pain or accidental needle pricks resulting in bleeding or infections from traditional needles, hypoglycemic shock, peripheral hyperinsulinemia, lipodystrophy, obesity, and macro- and microvascular complications (Yang et al., 2018; Zhao et al., 2022; Heinemann et al., 2023). After S.C. INS injection, only 20% of the injected dose reaches the target. The bioavailable fraction after oral INS is 1–2% (Collado-González et al., 2020). This is due to the hepatic first-pass metabolism resulting in ineffective control of BGLs. Moreover, <50% of chronic diabetic patients adhere to their injectable INS dosing regimen (Chen et al., 2022). It was estimated that 35% (37/105) of the new diabetic patients prescribed with S.C. INS delayed INS therapy due to injection phobia. Approximately, 80% of T2D patients (47/59) were afraid of needle pain and 52% (243/648) were unwilling to start INS therapy (Duncanson et al., 2021). Still today, after the centenary of injectable INS discovery in 1921, diabetic patients and healthcare workers are anticipating an alternative, personalized, pain-free, and self-administrable unit dosage form (Smith et al., 2022).

Microneedle (MN) is of micron-scale size ranging from 25 to 2000 μm in length (Xie et al., 2015; Zhao et al., 2022), 50–250 μm in width, and 1–25 μm in tip diameter and a surface area of up to 2000 mm^–2^ arranged as an array (Jin et al., 2018; Nagarkar et al., 2020). They have micrometer-scale projections that penetrate the thick stratum corneum and deposit the drug in the viable skin layer. The absorption of the drug occurs from the rich dermal microcirculation (Jana & Wadhwani, 2019). Insulin microneedles (INS-MNs) are capable of dissolving or releasing the peptide in the dermal layer within minutes (less wear time). Common post-application limitations include erythema, itching, and tenderness at the site (Smith et al., 2022). This was solved by researchers by carefully adjusting the needle height and selecting the biodegradable polymer with lesser wear time (Pere et al., 2018; Zhang et al., 2022). In addition, MN reduces the probability of microbial penetration and contamination in comparison to traditional needles (Le et al., 2022). MN insertion depth is a maximum of 900 μm but the S.C. needle pierces to 4–6 mm (up to 9000 μm) beneath the skin (Heinemann et al., 2023). Repeated INS injection administration with a hypodermic needle causes adipose tissue atrophy, skin thickening, nerve damage, tissue necrosis, and ulcerations at the injection site (Zhang et al., 2021). The INS-MNs are advantageous for the juvenile diabetic population as they are the ones predisposed to trypanophobia. In a research survey on MN influenza vaccination patches fabricated with 50% polyvinyl alcohol (PVA) and 50% sucrose (phase 3 clinical trial), 99% of the needles penetrated the outer skin of the participants, and 70% of MNs dissolved after insertion with a wear time of 20 min. The percentage of participants administering the MN patch properly and the number of participants preferring MN over traditional needle vaccination were 86% and 93%, respectively (Arya et al., 2017). To date, Soluvia^®^ and Micronjet^®^ MN-based therapeutics delivery systems were approved by U.S. FDA (Le et al., 2022).

Biodegradable and biocompatible polymers are used in the dissolving and swellable hydrogel-based polymeric MNs. Biopolymers are in demand for MN fabrication as they are natural, sustainable, inexpensive, biocompatible, safe, and nontoxic. The biodegradable polymeric MNs are formulated in ambient conditions under vacuum or pressure (mold filling and drying) without any heating steps or the use of organic solvents or UV irradiation (Sullivan et al., 2008). Hence, the heat-sensitive peptides or growth factors not being denatured during the fabrication step (Chellathurai et al., 2021; Zhang et al., 2021). Dissolving MNs are used for providing bolus INS shots for immediate release. Hydrogel MNs made from cross-linked polymers are used for maintaining steady-state plasma levels of INS for a prolonged duration (Mbituyimana et al., 2022). Hydrogel MNs are capable of wicking interstitial fluid to swell and biodegrade slowly in the body thereby releasing the entrapped INS from the cross-linked network. Moreover, the dose requirement of INS through MN delivery is even lesser than the S.C. injection (Fonseca et al., 2020) and oral delivery (Jin et al., 2018). Thus, the limitation of mold capacity is not of much concern and could be overruled (Sharma et al., 2019; Detamornrat et al., 2022; Noh et al., 2022). The barriers that hinder the clinical translation of INS-MNs are dosing variability among diabetics and dose-adjusting techniques. To overcome this issue, glucose-responsive MNs (artificial pancreas INS system) and a combination scheme of glucose-responsive INS-nano particulates entrapped in the MN cavity were researched to release INS based on the biochemical trigger (elevated glucose amount) in the blood (Zhang et al., 2020).

In this review, dissolvable/biodegradable and hydrogel-forming swellable INS-MNs have been explored about their polymer choice, fabrication, and significance in effective INS delivery. Few researchers have reviewed all types of polymeric transdermal MNs (including solid, coated, and hollow) (Jin et al., 2018) and polymers used in transdermal MNs (Chen et al., 2018). Moreover, we have projected and highlighted the implications and significance of INS-MNs for intra-oral, implantable, and gastrointestinal (GI)-mucosa-targeted INS delivery. Also, recent publications (mainly, 2018–2023) on INS-loaded biodegradable MN-based delivery systems were critically evaluated to elucidate the progress in this field. Biodegradable MN-based INS delivery systems were overviewed with their process parameters, advantages, limitations, and the novel approaches carried out for optimal drug release from the system for better INS bioavailability. The inclusion criteria for this review are biodegradable MNs for transdermal and oral INS delivery fabricated using simple, low-heat methods, and the researched devices that utilize biodissolvable micron-sized needles to pierce through the GI mucosa for INS delivery. The self-administrable S.C. INS-MN injections, wearable and sensor-based MN-based devices for diabetic management, bioavailability enhancement of INS other than MN technology, UV irradiation and high-heat involving MN fabrication methods, solid, coated, and hollow MNs, and the non-biodegradable and non-biodissolvable polymeric MNs were excluded. The search strings used in Google Scholar/PubMed were ‘biodegradable polymeric insulin microneedles’, OR ‘dissolvable insulin microneedles’, OR ‘transdermal insulin microneedles’, OR ‘oral insulin microneedle devices’, OR ‘hydrogel-based insulin microneedles, OR ‘bio-inspired insulin microneedle devices’, OR ‘gastrointestinal insulin delivery AND “microneedles”’, OR glucose-responsive insulin microneedles, OR ‘insulin micromotors AND “microneedles”’, insulin microneedles OR ‘microneedle insulin devices’, insulin microneedles NOT ‘glucose monitoring’.

## 2. Pharmaceutical significance of encapsulating and delivering insulin as a biodegradable INS-MN delivery system

It is essential to understand the superiority of MN delivery approaches for INS over the highly researched oral and existing S.C. INS injection. The comparison of advantages and limitations of INS-MNs, oral INS and injectable INS is given in Table 1.

**Table 1.** Oral, microneedle, and subcutaneous insulin comparison (Jin et al., 2018; Zhao et al., 2022; Chellathurai et al. 2023).

Parameters	Oral insulin	Microneedle insulin	Injection insulin
Patient acceptability	Convenience and patient compliance	Self-administrable, negligible pain and lesser side-effects	Local injury and pain Hypoglycemic shock Lesser compliance
Pharmacological factors	Mimics pancreatic insulin pathway	The absence of proteolytic enzymes and harsh pH in the skin or mucosal layers meant for INS-MN insertion renders INS safe and effective to be administered	Only 20% reach the target due to the shorter half-life and faster metabolism in the liver and kidney
Pharmacokinetic factors	Low absorption and low bioavailability (1-2%)	Faster onset of action Maximum drug utilization and higher bioavailability	100% bioavailable Repeated multiple dosing required to maintain normoglycemia
Hepatic first-pass effect	Hepatic first-pass effect	Bypasses metabolic pathway	Faster metabolism
Degradation threshold	Easily degraded in the acidic stomach	Could be protected from degradation, by loading as core–shell or coated insulin nanoparticles	Shorter half-lives of insulin require modifications to overcome the same
Pharmaceutical parameter	Need for permeation enhancers Larger dose requirement	Less dose requirement Increased stability of insulin (dry state) Inter- and intra-personal variability makes dose adjustment difficult	Stabilizers and modifiers requirement. Larger doses and repeated administration are necessary

The INS-MNs are the minimally invasive, easy-to-handle, self-administrable personalized dosage form that reduces healthcare costs and thus the diabetic patients’ economic burden (Jin et al., 2018). Neither heating nor organic solvent usage is necessary for the fabrication or storage of INS-MNs by micro-molding method. So, the formulated INS-MN system can preserve the conformational stability of the peptide hormone during the casting process and in the course of storage. Insulin, after incorporation into the polymer matrix, forms molecular interactions. And, when molded into the MN, the movement of INS molecules is suppressed. Thus, reducing the chances of aggregation, phase separation, or recrystallization. It was observed that the powdered INS in MN was 100% stable at −20 °C as there was no residual water content. At a temperature of 25 °C, dry INS incorporated into the MN was 93.3 ± 3.8% stable for about 8 weeks (Kim et al., 2020). This might overcome the necessity of cold-chain storage as the drug is in the dry form without any interaction with water molecules and the mobility of INS was rendered null within the system (Fonseca et al., 2020; Kim et al., 2020; Cao et al., 2022). Still long-term stability analysis >8 weeks is required. It was also observed that a stabilizing shell was formed by the incorporated polymers or sugars to replace the removed water molecules of the INS to enhance the stability of the loaded INS (Smith et al., 2022).

Since the dermal layer is hydrophilic and has a large number of blood capillaries, INS could easily diffuse through the dermis and get absorbed into the systemic circulation after MN insertions. Also, negligible pain is felt as the MNs do not stimulate the nerves in the lower dermal region (Fonseca et al., 2020). Controlled release of INS is achievable for an extended duration based on the biodegradation of the cross-linked polymer used (Ye et al., 2023). Thus, maximum drug utilization from the incorporated low dose is possible to maintain normal BGLs with less glucose fluctuation (Zhao et al., 2022). Since the drug released from the MNs *in situ* is in minute quantities, adverse effects like hyperinsulinemia, hypoglycemic shock, and subsequent risks of concentration-related side effects are significantly reduced (Zhang et al., 2021; Zhao et al., 2022).

Faster absorption and rapid glucose-lowering effect were reported with GI delivery of INS-MNs via intragastric devices. These advantages create new pharmaceutical research avenues for INS delivery through non-parenteral routes. The MNs were reported to cause little damage to the applied sites and the perforations formed were able to recover within a few minutes to 24 h (Chen et al., 2018). Transdermal and non-transdermal MN delivery routes like buccal, sublingual, etc. could bypass the hepatic circulation and GI degradation. Hence, a comparatively lower dose (0.2 IU INS/kg) than S.C. and oral INS is sufficient to produce an equitable response (Fonseca et al., 2020). Moreover, a simple fabrication process with the flexibility of drug loading (tip-loading, needle-layer loading, and complete needle cavity loading as in implants) is advantageous for in-expensive mass-production and rapid scale-up (Kim et al., 2020).

## 3. Design principles for biodegradable/dissolvable polymeric insulin microneedles

### 3.1. Characteristics of INS supporting MN delivery

Insulin is a large molecular weight potent peptide hormone (5808 Da) with a short half-life (3–10 min). Other characteristics of INS are listed in the review paper by Chellathurai et al. (2023). It is a polar hydrophilic peptide (Chen et al., 2018) with a *log P* value of −1.61 (Goo et al., 2022), and a melting point (m.p.) around 65 °C (stable zinc-INS hexamer has a m.p. of 84 °C). Insulin gets unfolded at a temperature of 50 °C (Kaufmann et al., 2021). It consists of two different A and B peptide chains with 51 amino acids. It is of about 151 nm average diameter in water (Wang et al., 2019). Its isoelectric point (pI) is 5.3. It has a charge of −2 to −6 in pH 7–11. The approximate INS dose of a T1D patient is given as 0.5 units/kg (36 units for a 72 kg person) (Kim et al., 2020) or 16 IU/day (Wang et al., 2019). In 1 mg of recombinant human INS, 27 USP IU of INS is present. The loading doses reported in many INS-MN devices were in the acceptable range of INS dose (0.1–20 IU) with better cumulative release results to meet diabetic patients’ dose requirements (Cao et al., 2022). To increase the transport across the hydrophobic mucosa, it is necessary to increase its lipophilicity. Also, the hormone should be well-protected in the delivery device until delivered at any intended sites. Due to the peptide’s shorter lives, it would be better to pre-program the INS release for a longer duration from the delivery device. The INS release could be destined as per the patient’s need, as a closed-loop glucose-responsive self-regulated system mimicking pancreatic INS release based on the elevated biochemical trigger glucose (Chen et al., 2020). Another approach is based on timed biodegradation/erosion within the body to release INS from every layer slowly and steadily from the alternative INS and biodegradable polymer layer-by-layer coated nanoparticles (NPs) (Zhang et al., 2021). The MN design characteristics like morphology and dimensions, the type of polymer used, and the manufacturing process involved in the fabrication affect the permeation and release kinetics of the polymer MNs. In a nutshell, the designing and evaluation of the INS-MN delivery system should be done by keeping the end-user in mind and formulating a patient-centered dosage form.

### 3.2. Polymer selection for biodissolvable/biodegradable insulin microneedle formulation

**Figure 1.**
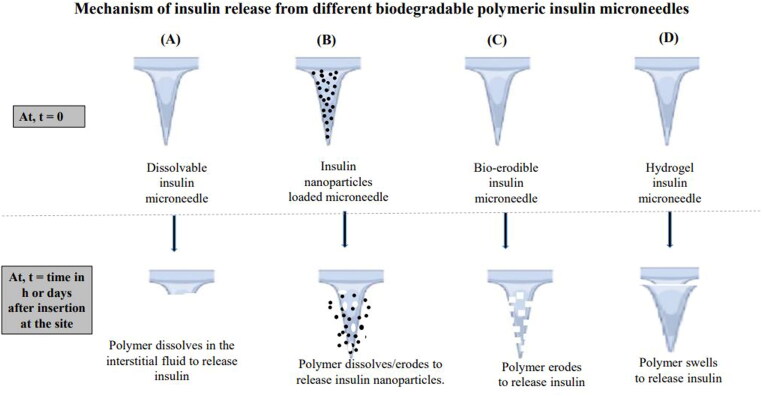
Mechanism of insulin release from the MNs. Author’s renderings, inspired from the Figure 3(b) in Le et al. (2022).

The polymer selection for MN fabrication is important as the polymer choice dictates the needle thickness, needle density, insertion effectiveness, and complete payload release and in turn, determines the consistency of dose delivery. Polymer characteristics and their impact on MNs are summarized in Table 2. The mechanism of INS release from different types of polymeric INS-MNs is shown in Figure 1 (Chellathurai et al., 2021).

**Table 2.** Polymer characteristics and their impact on MN formation.

Polymer characteristics	Impact on MN formation	Significance	Reference
Polymer concentration Highly viscous solution: Low viscous solution:	The viscosity of the polymer solution increases with an increase in the polymer concentration. The viscous nature prevents the solution from reaching the sharp tip of the MN mold and incomplete MN is formed. They get easily spun off from the microneedle cavities resulting in incomplete MN formation. Hence an optimal concentration range should be optimized for the fabrication.	Insulin release characteristics are dependent on the amount of polymer used in the fabrication. The higher the concentration, the longer the time for insulin to be bioavailable. The bioerosion of polymer takes longer than low-concentration MNs.	Chellathurai et al. (2021)
Solubility and degree of dissolution	MN tips made of easily soluble polymers disintegrate quickly to release insulin. MNs made of supramolecular cross-linked polymers get swollen to release INS for a prolonged duration of >12 h.	The polymer characteristics can be modulated to regulate drug delivery by cross-linking. A higher degree of covalent crosslinking lowers the degradation rate of the polymer matrix.	Ye et al. (2023)
Polymeric molecular weight (MW)	Low MW polymer showed greater rupture displacement and high rupture force than high MW polymer. Rupture stress required was similar for low and high molecular weight polymeric MNs.	Polymers of MW <66 kDa are drained into the dermal blood capillaries with a minimal amount reaching dermal lymphatics before reaching the systemic circulation. Polymers of MW <60 kDa could be excreted via kidneys after glomerular filtration. The maximum deformation capacity of the MN before rupturing is the rupture displacement that was affected by the contact area at rupture.	Du et al. (2021) and Smith et al. (2022)
Elastic modulus <1 GPa	Buckling fracture and failure before penetrating the skin. Fillers and other agents added too increase the elastic modulus of the polymer.	Fracture strength is directly related to polymer elastic modulus and needle base diameter and inversely related to MN length.	Zhao et al. (2022)
Polymer crystallinity (for base-layer)	Heat-treated PVA of high MW (89–98 kDa) has greater aqueous stability and excellent biocompatibility	A long-lasting MN patch was formed that is durable for a week of insulin administration.	Chen et al. (2020)
Sucrose addition with polymer	Helps in forming stronger needles.	Stabilizes the encapsulated insulin	Zhang et al. (2021)
Incorporation of drugs into the polymeric MNs	Reduces Young’s modulus of the MN formed. Hence, decreases the mechanical strength of the needle.	Decreases the penetration capacity.	Du et al. (2021)
Young’s modulus	MN made of high molecular weight polymer showed a higher Young’s modulus than low molecular weight polymer.	Young’s modulus reflects the stiffness of the MN and relates to the tensile strength of the polymer but this relationship varies significantly for different polymers.	Du et al. (2021)

In dissolvable INS-MNs, INS is encapsulated within the MN matrix of water-soluble polymers. After insertion, the water-soluble polymer gets dissolved in the body fluid to release INS (Figure 1(A)). Water soluble polysaccharides like hydroxyl propyl methyl cellulose (HPMC) (Abramson et al., 2019), carboxymethyl cellulose (CMC) (Kim et al., 2022), dextran (Ye et al., 2023), alginate (Yu et al., 2017), starch and gelatin blend (Chen et al., 2018; Jana et al., 2022) were used for dissolving MN fabrication as no biohazardous sharp residues are left beneath the skin. Other polymers like poly-γ-glutamic acid (γ-PGA) (Chen et al., 2015), polyvinyl pyrrolidone–polyvinyl alcohol (PVP–PVA), poly(methyl vinyl ether-co-maleic anhydride) (PMVE/MA) (Garland et al., 2012), and PVA (Zhang et al., 2021; Liu et al., 2022) were also used in dissolvable MN fabrication. Sugars like trehalose, xylitol, and mannitol were mixed with INS and coated by inkjet printing to produce uniform, reproducible, and precise coats on the MNs made of dental SG resin or photo-sensitive FDA-approved class 1 resin (Pere et al., 2018; Economidou et al., 2019). These coats dissolve rapidly within 30 min and the INS was found to be stable as the coats were in the dry state. It was found that the combination of sucrose with PVA in water (6:8:15) enhanced the strength and quality of the MNs formed (Zhang et al., 2021). Dissolving MNs are usually administered for bolus or mealtime dose as the MN shafts dissolve faster to release the drug (Zhao et al., 2022; Bauleth-Ramos et al., 2023). If required to achieve sustained release using hydrophilic polymers, then we should select either a higher concentration of polymer or choose a high molecular weight or physically (freeze-thawing) (Chen et al., 2021) or covalently crosslink the dissolvable hydrogel polymer.

When the macromolecular drug recombinant human keratinocyte growth factor (Chellathurai et al., 2021) alone was entrapped in the microcavities, a rapid release was achieved. To attain a prolonged or controlled concentration of peptide drugs in the body with long-term hypoglycemic action, nanometrics like liposomes (Qu et al., 2023), nanovesicles (Chen et al., 2022), layered cargo NPs (Verma et al., 2016; Zhang et al., 2020; Dawud & Abu Ammar, 2023), or double encapsulation of cargo could be done (Chen et al., 2020; Jiang et al., 2022). The kinetics of INS delivery from these combination scheme devices are based on the biodegradation of the NPs’ coat or matrix, and hence sustained delivery is possible (Figure 1(B)).

Polymers used are polysaccharide chitosan (Zhang et al., 2020; Wang et al., 2023), γ-PGA (Chen et al., 2015), alginates (Yu et al., 2017), calcium cross-linked alginate/maltose (Zhang et al., 2018), hyaluronate (Chen et al., 2022), pullulan (Fonseca et al., 2020), methacrylic modified hyaluronic acid (HA) (Zong et al., 2022). These are the biocompatible materials resembling the composition of the human extracellular matrix (chitosan, HA, and dextrin) and are biodegraded in the body without leaving any toxic residues. Synthetic biocompatible polymers, PVA, polyvinyl pyrrolidone (PVP) (Chen et al., 2015), polylysine (PL) (Shen et al., 2021), PL: guar gum blend (Lu et al., 2023), polylactic acid (PLA) (Chen et al., 2018), polyglycolic acid (PGA) layer-by-layer coated MN (Wang et al., 2020), poly(lactic-co-glycolic) acid (PLGA) (Li et al., 2019), genipin cross-linked gelatin (Chen et al., 2018), polyallylamine and PVA (Chen et al., 2021), and cross-linked poly(acrylamide-co-acrylic acid) (Qiu et al., 2012) were used for biodegradable MNs fabrication. Synthetic polymers too are biodegradable and after application onto the skin degraded to smaller lactic and glycolic acids (PLA, PGA, and PLGA) and get excreted from the body. Other polymers like polypeptide protein polymers gelatin and silk fibroin (Wang et al., 2019; Zhu et al., 2020), and biodegradable bio ceramics like gelatin-hydroxyapatite (Yu et al., 2017) were also used. Polymers used in INS-MNs are of great concern as the MNs are deposited within the skin from one minute to an hour for effective dissolution. Hence, biodegradable and regulatory-approved polymers should be used, which degrades by enzymatic hydrolysis into nontoxic agents (Figure 1(C)).

Swellable hydrogel INS-MNs can yield constant and reproducible INS release from the system (Figure 1(D)). The biodegradable water-insoluble polymer could be physically (freeze-thawing) or supra molecularly cross-linked (Ye et al., 2023) to dry hydrogel and is used for fabricating hydrogel MNs (Mbituyimana et al., 2022). After administration into the dermal layer, the hydrogel imbibes the interstitial fluid, expands and produces conduits for INS release through the swollen hydrogel networks.

### 3.3. Fabrication techniques

Micro-mold casting technique is the widely employed method to prepare polymer MNs. The polydimethylsiloxane (PDMS) molds or silicone molds are used to prepare MNs. The molds are reusable 100 times, and hence cost-effective (Donnelly et al., 2010). The porous molds are air-permeable but water-impermeable (Zhang et al., 2021). This breathable property helps the MN forming-drug-polymer solution in the mold to reach the tips of the micro-cavities on applying a vacuum and aids drying as air permeates through them. Approximately, a 10 × 10, 750 μm depth, 300 μm base, and 500 μm tip-to-tip spacing mold have a volume capacity of 2 μL per needle (Zhang et al., 2021). Since INS is sensitive and heat-labile, aqueous soluble polymers are chosen for the fabrication. The polymer solution (along with stabilizer and other excipients) will be filtrated through a 0.22 μm nylon membrane filter to remove any dead and viable contaminants. The calculated quantity of INS will be dissolved in its solvent (0.01 N HCl, pH 2.8, solvent filtered through 0.22 μm filter) and will be mixed with the previously filtered polymer–excipient solution. This drug–polymer–excipient mixture will be pipetted using micropipettes onto the molds or sprayed onto the mold microcavities. Air voids are eliminated by reduced vacuum or centrifugation. The filled MN mold was kept for drying in ambient conditions in a controlled environment in a class 100 laminar flow workstation (Chellathurai et al., 2021). After curing and de-molding, the MNs formed will be evaluated.

**Figure 2.**
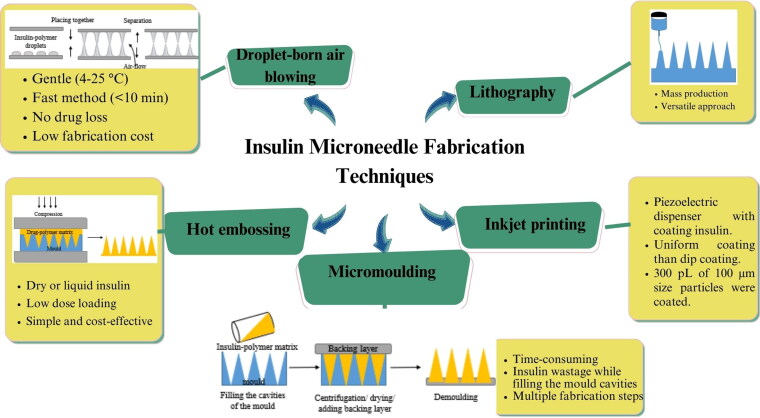
Insulin microneedle fabrication techniques, its advantages and disadvantages. Hot embossing: adapted and reproduced with permission from Li et al. (2019), Copyright (2019), Elsevier. Micromoulding, droplet-born air blowing and lithography: Adapted and reproduced from Sartawi et al. (2022) CC-BY (2022), Elsevier. https://s100.copyright.com/AppDispatchServlet?publisherName=ELS&contentID=S0168365922003169&orderBeanReset=true (accessed on 16 November 2023).

Other techniques of MN fabrication include droplet-born air blowing (DAB) (Xie et al., 2015) of micro-molding, drawing lithography (Pere et al., 2018; Kim et al., 2022), inkjet printing (Pere et al., 2018), and hot embossing (Li et al., 2019). The review article by Zhao et al. describes the fabrication techniques used for heat-sensitive drug-MN production (Zhao et al., 2022). In the DAB method, the drug–polymer droplet is molded into a MN by air-blowing. The dose in the droplet is controlled by modulating the concentration of the INS–polymer matrix or by modifying the droplet volume. Insulin was found to be stable and maintained its biological activity (Kim et al., 2013). The advantages and disadvantages of these methods concerning INS loading are recorded in Figure 2.

## 4. Factors considered for transdermal microneedle-based insulin delivery

### 4.1. Factors related to MN design

**Figure 3.**
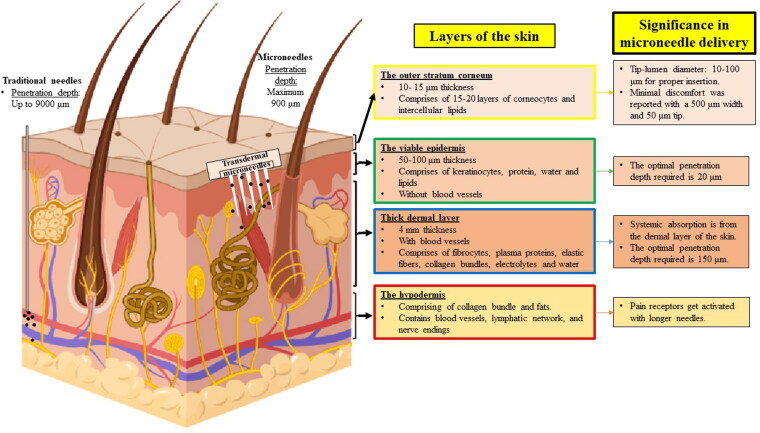
Layers of skin and their significance in INS-MN delivery. Author’s renderings, inspired from Figure 1 in Jamaledin et al. (2020).

The length of the MN should be optimal to minimize tissue damage while piercing and to minimize pain without reaching the nerve endings beneath the skin. The length, base width, and tip diameter should be predetermined for appropriate penetration depth. Human skin consists of four layers and the layer size varies with different sites (Chen et al., 2018) (Figure 3). When the needle diameter increases, the penetration force increases (Caffarel-Salvador et al., 2021). The fracture force of >0.1 N/needle could pierce through the skin smoothly (Ita, 2017).

The mechanical strength of the MN is directly related to Young’s modulus and fracture toughness of the polymers. Young’s modulus of a few biodegradable polymers is tabulated in Table 3. Greater the mechanical strength better the insertion capability, deeper the penetration and higher the drug release. The reported limitation of dissolvable MN was their poor mechanical property and their low dose capacities (Zhao et al., 2022). Mechanical strength could be increased by (1) increasing the concentration of the needle-forming polymer or combining polymers in the fabrication process. (2) When an additional step of vacuum freeze-drying was incorporated, the water-containing polymers tend to become hard leading to effective stratum corneum penetration (Zhang et al., 2021). (3) Increasing the wall thickness, wall angle, smaller tips, and reduced aspect ratio with circular-shaped MNs were observed to have greater mechanical strength (Jin et al., 2018; Zhao et al., 2022). (4) Crosslinking of polymers could be done to enhance the strength of the MN formed. Freeze-thawing, forming supramolecular complexes, and covalent bonding were the cross-linking techniques used. Covalent crosslinking with chemical linkers or *in situ* polymerization yields toxic by-products introduction into the MN device. So, phenylboronic acid (PBA) having diol bonds was cross-linked within polymeric MNs to perform a dual role for both network cross-linking and glucose sensing (Ye et al., 2022). Here, dynamic-covalent polymer hydrogels with shear-thinning ability and self-healing properties filled the cavities and recovered to their original mechanical strength through bond rearrangements (Ye et al., 2022). (5) Air-bubble entrapment (for high MW concentrated polymers) should be avoided and if present should be removed by applying a vacuum as it may decrease the strength of the needle. It was also found that storing the prepared polymer solution undisturbed overnight helps in stabilizing and forming viscous solution without any air bubbles for MN fabrication.

**Table 3.** Young’s modulus of biodegradable polymers (El Miri et al., 2015; Naser et al., 2021; Sachan et al., 2021; Zhang et al., 2021; Darge et al., 2022; Ranakoti et al., 2022).

Biodegradable polymer for MNs	Molecular weight (kDa)	Young’s modulus *E* (GPa)
Sodium carboxymethyl cellulose (CMC)	90	1.066
Polylactic acid (PLA)	160	0.35–3.5
Poly(l-lactic acid) (PLLA)	120	2.7–4.14
Poly(l-lactide-co-glycolide) (PLGA)	30–66	3–6.04
Poly(d,l-lactic acid) (PDLLA)	100	1–3.45
Poly(glycolide) (PGA)	>30	6.0–7.0
PDLLA/PGA (50:50)	45	1–4.34
PDLLA/PGA (75/25)	66–107	1.38–4.13
Poly(caprolactone)	15	0.21–0.44
Chitosan	135	18.8

The fracture force of the needle should be always higher than the evaluated insertion force for the MNs fabricated. The stronger the MN, the better the penetration without bending or breaking while insertion. Also, it was reported that the strength of the unloaded tip was higher than the INS-loaded tip (Ghosh et al., 2022). Drug loading weakens the MN strength (Tuan-Mahmood et al., 2013; Du et al., 2021). However, the dissolving MNs with drug-loaded NPs were observed to be of acceptable hardness and insertion capabilities (Dawud & Abu Ammar, 2023).

As the MNs pierce the outer skin, sterility of the formulation is essential. INS is a thermo-labile, radiation-labile, and sensitive peptide. So, to maintain the sterility of the MNs the preparation should be carried out in an aseptic-controlled environment and the polymer matrix should be filtered through an aseptic 0.22 μm polystyrene filter membrane to remove the viable and dead micro-organisms (Chellathurai et al., 2021; Ghosh et al., 2022). The storage of the prepared MNs has to be considered as chitosan, PVA, gelatin, and HA MNs weaken and dissolve when the humidity is above 80% and will result in insertion failure. High temperatures denature INS. So, the stability of INS in various temperatures and humidity should be studied. Desiccants were used to protect the MNs from humidity and appropriate packaging to eliminate dust and other contaminants is necessary. Overall, the stability and sterility of the INS-MNs should be preserved until use by the patients.

### 4.2. Factors related to MN application and insulin release

The proportion of needles inserted efficiently beneath the skin demonstrates insertion efficiency and in turn the amount of dose delivered. This gives the data on the controlled release behavior of the system for maintaining a consistently reproducible INS release for achieving steady blood glucose concentration. Incomplete insertion leads to incomplete drug delivery. When a low dose is delivered, drug wastage and less therapeutic response could be seen (Zhang et al., 2021). Skin thickness varies for different age groups, ethnic groups, skin types, different body sites, genders, body mass index, and the presence of skin diseases. The wound healing ability, elasticity, and moisture content of the skin dictates the MN insertion and the micropore closure kinetics. For the juvenile and skin disease population, the skin would be of thinner corneocytes, weaker epidermis, thinner stratum corneum, lesser melanin, and moisture, etc. (Alshammari et al., 2022). Hence, the MN for these groups should be designed carefully. The force that is required for insertion using a human finger is 12 N (Kamal et al., 2020; Kim et al., 2022) and the thumb is 20 N (Li et al., 2019). The optimal force required for 20 μm and 24 μm tip diameter MN was about 0.15 N per needle (Yu et al., 2017; Tong et al., 2018). Also, insertion depth would be increased by having larger needle-to-needle spacing and using higher insertion force. If the spacing between the needles is small, then the ‘bed of needles’ effect with reduced penetration will be seen. To reduce the risk of MN fracture during tissue penetration, the tip-diameter, length and width ratio and angle of insertion are taken into account. The needle tip of 5 μm produced smoother penetration and always the tips should be <15 μm for easier insertion. Generally, a tip width of <50 μm and base width of 500 μm were considered to cause lower pain sensations while insertion (Bonfante et al., 2020).

Chen et al. selected a tip angle of 15° that allowed easy and effective penetration of 700 μm length MN without disturbing the nerves and blood vessels (Chen et al., 2020). However, up to 30° top angle was acceptable for easy penetration (Bonfante et al., 2020). The mode of compression force applied onto the MN patch is responsible for the maximum insertion of MNs in the skin layers. However, the force applied by different patients varies with uneven force distribution on the patch leading to needle fracture. Hence, actuators with necessary activation forces should be designed.

After the application of a transdermal MN patch, while stretching or twisting the applied skin, there are chances for the deformation of the patch system that might challenge the performance of the system. Hence, research on patchless transdermal MN delivery systems has evolved. Few approaches researched were given. (1) Introduction of air bubble or creation of air bubbles due to the effervescence of CO_2_ gas between the needle head and the backing layer. They incorporated sodium bicarbonate and citric acid between the needle tips and the supporting base layer. After the MN insertion, the interstitial fluid comes in contact with the salts to dissolve and leading to the evolution of CO_2_ gas for the subsequent detachment of tips (Zhang et al., 2020). (2) Use of dissolving base film (CMC) and micropillars (Kim et al., 2022). (3) Introducing a thermo-responsive layer between the needle and base layer (Zhang et al., 2020). (4) Fabrication of detachable polymeric MNs (Ye et al., 2023). Transdermal INS-MN designing aspects and the reported outcomes are summarized in Table 4.

**Table 4.** Transdermal insulin microneedles’ design characteristics and the research findings.

Transdermal biodegradable polymeric INS MNs	Polymer characteristics/type/fabrication method	MNs geometry	Outcome and its impact on insulin delivery	References
Pullulan (24%) with 12.4 IU insulin/MN patch (dose in each patch for 62 kg adult)	Nontoxic, aqueous soluble, low viscous solution, film-forming, good adhesiveness mechanical strength, non-cytotoxic Dissolving MNs Solvent casting micro-molding technique	Density of solution: 1.07 g cm^–3^ Solution viscosity: 1148 mPa s MN length 500 ± 14 μm Base width: 201 ± 3 μm Tip-to-tip distance: 500 ± 1 μm Aspect ratio: 2.45 ± 0.07 Tips: 3–15 μm Height reduction from mother mold: 6–11%	Smooth and crack-free MN surface. Penetration depth: between 40 and 381 μm *In vitro* release: 87% released by 2 h Temperature stability: stable for 1 month at 4, 20, and 40 °C without secondary structure conformational changes	Fonseca et al. (2020)
Polyvinyl alcohol (PVA) MW 6000 Da:sucrose (stabilizer):water (8:6:15)	Biocompatible, hydrophilic, good mechanical properties and processability. Dissolving MNs Solvent casting micro-molding technique	Viscosity: 3700 mPa s Failure force: 0.3 N/needle Insertion force: 0.2 N/needle Insulin loading: 5.02 μg insulin/patch Insulin left over after insertion (the attached portion with base plate): 0.43 μg insulin/patch Drug delivery efficiency: 92%	*In vitro* dissolution of MNs (into porcine cadaver skin) in interstitial fluid: within 2 min. *In vivo* duration of action in diabetic mice: 3 h for INS-MNs and 2 h for S.C. injection.	Zhang et al. (2021)
PVA heat treated (MW 89–98 kDa) for the base layer. Glucose responsive hydrogel for base layer needle tips: Boronate compound with N-isopropylacrylamide (NIPAA_m_ or N,N-dimethyl-acrylamide (DMA) for controlling the hydrophilic and hydrophobic balance	Excellent biocompatibility and aqueous stability after heat-treated crystallization. Timed-dependent degradation of −3.2 ± 2.1% Dissolving hydrogel MNs	Mechanical strength of two-layer MNs: 0.106 N/needle The minimum force required for insertion without breaking is 0.06 N/needle	Homogeneous distribution of insulin. Hydrogel possessed glucose-dependent volume change for insulin release in hyperglycemia and was silenced when glucose concentration decreased. Negligible temperature dependency for insulin release. Long-lasting release of insulin for several days (78 h).	Chen et al. (2020)
Proline:silk fibroin (3:10)	Biocompatible, biodegradable, better mechanical properties. Degradable hydrogel MNs	15 × 15 needle arrays Insulin loading: 1.25 mg (≈33.75 IU) Proline-treated fibroin: greater swelling degree (80–200%) Methanol-treated fibroin: 30% swelling degree Proline amount in fibroin MN is inversely related to the pore size created by the same MN. Fracture strength >0.5 N	Needles are nontoxic and exhibited sustained release for 60 h with a cumulative release of 67.9%. Unmodified fibroin random coils exhibited rapid release for 24 h. Proline changed the crystal structure of the fibroin and thus the swelling degree increased to 80%. The controlled effect was observed due to slow dissolution. Microneedle pore diameters formed were 50 nm and 100 nm.	Wang et al. (2019)
Glucose-responsive cross-linked methacrylated hyaluronic acid INS-MNs loaded with red blood cells or liposomes containing glucose transporters (GLUTs) bound with glucosamine insulin (Glu-INS).	Biocompatible and biodegradable. Micro-molding technique.	15 × 15 needle arrays Conical needle Tip diameter: 10 μm Base: 300 μm Height: 600 μm Mechanical strength per needle: 0.55 N Insertion strength: 0.1 N Insulin loading in RBC-INS vesicles: 0.5 wt%	In hyperglycemia, the elevated glucose concentration competitively binds with GLUT to release Glu-INS for regulating BGLs. In diabetic rats, normoglycemia was achieved within 1 h and maintained at 200 mg/dL for over 5 h which was higher than injections. Sustained release and glucose-responsive release achieved.	Chen et al. (2022)
Glucose-responsive 3-aminophenyl boronic acid-modified-alginate-hyaluronate INS-MNs	Biodegradable, non-cytotoxic, sustainable	10 × 10 needle arrays Tip diameter: 10 μm Base: 300 μm Height: 700 μm Mechanical strength per needle: 0.37 N Insertion strength: 0.098 N Insulin loading: 10 IU	Alginate-HA crosslinking in the presence of Ca^2+^ ions increased the microneedle strength. In S.D. rats, euglycemia was maintained for around 6 h with MNs and around 4 h with S.C. injection. The *T*_max_ was 3 h for the MNs which is higher than the injection proving sustained release kinetics. Pharmacological activity of 90.5% Bioavailability was 92.9 %	Yu et al. (2017)
Base layer: CMC film Needle: HA 20% droplets Micropillars for finger insertion force	Centrifugal lithography (202 g force) Implantable patchless dissolving MNs.	5 × 5; 25 needles Diameter: 350 μm Height: 1.5 mm Punching force using CMC film using force analyzer trigger: 4.35–11.05 N Micropillar punching force on film: 4.32–11.05 N Insulin loading: 0.1 IU	No residual base layer leftovers containing INS in the array holder as the micropillars helped to push the entire needle heads within the implanted site. The punching force was found to be increased with micropillar diameters and polymer concentration. Higher INS plasma levels were found with patchless film triggered applicator than with dissolving patch MNs. Bioavailability of 93%	Kim et al. (2022)
Insulin:mannitol, insulin:trehalose, insulin:xylitol (5:1 ratio for all) MN: dental SG resin	Dissolving coats on the microneedle Stereolithography and Inkjet printing	Insulin loading: 10 IU (350 μg/array) Base: 1000 μm Height: 1000 μm	Insulin with xylitol maintained the native form of insulin. Stable for 30 days at 2–8 °C. Rapid insulin release (>90%) within 30 min.	Pere et al. (2018)
CMC combined with polyethylene glycol macromer terminally modified with Brooker’s merocyanine (BM) added with cucurbit[8]uril (PEG8a-BM⊂CB[8]) for supramolecular hydrogel formation (1:1) combined with different mechanical biopolymers like HA and dextran.	Dissolving MN with good strength, solubility and mechanical properties. Hydrogel-based and biodegradable. One-step fill and dry fabrication.	CMC tensile strength: 28 MPa Fracture force: 0.5 N/Needle Base: 300 μm Height: 600 μm Tip-to-tip spacing: 600 μm	Covalently linked hydrogels could not retain in the skin layers and peeled off with the patch. Supramolecular hydrogels swell and get detached within the skin layers for controlled and prolonged release. Supramolecular gels release rate of INS is 52 h and covalent hydrogels have a release rate of 96 h. Could release insulin for nearly 12 h and maintained 150–200 mg/dL of BGLs. <5 min skin application before needle detachment.	Ye et al. (2023)
Poly-γ-glutamic acid (γ-PGA) MN with PVA/PVP supporting structures.	Nontoxic, biodegradable, and non-immunogenic. Hydrogel-based biodegradable MNs.	9 × 9 needle arrays Base: 300 μm Height: 600 μm Insulin diffusion depth: 800 μm after 4 min of post-insertion Insulin loading: 0.20 ± 0.02 IU per patch	>50% of γ-PGA retained its strength after 55 % RH exposure for 3 days. More than 90% of insulin is stable at different storage conditions.	Chen et al. (2015)
Gelatin and hydroxyapatite (Hap)	Low cytotoxicity, biodegradable, chemically stable, biocompatible, and high compressible strength Molding technique.	8 × 10 needle arrays Base: 150 μm Height: 900 μm INS loading: 5, 10, and 20 IU	Crystalline Hap formation was hindered by gelatin addition. Higher INS plasma circulation levels than S.C. injection in rat models.	Yu et al. (2017)
Polylactic co-glycolic acid powders 60–130 kDa.	Biodegradable with a porosity of 20.1%. could load dry or liquid insulin into the pores. PLGA porous MNs prepared by hot embossing. Drug loading by dipping the tips in liquid or dry drugs.	12 × 12 needle arrays Base: 450 μm Height: 500 μm Tip diameter: 25 μm Tip loading region: 0–240 μm Insertion force: 22 mN Minimum energy for penetration: 23 μJ	INS loaded into the pores of PLGA MNs. The capacity that could be absorbed by the pores was 0.31 μL to 1 μL. Bendable and penetrated rat skin with a force of 5 N.	Li et al. (2019)

### 4.3. Factors considered for non-transdermal microneedle-based insulin delivery

**Figure 4.**
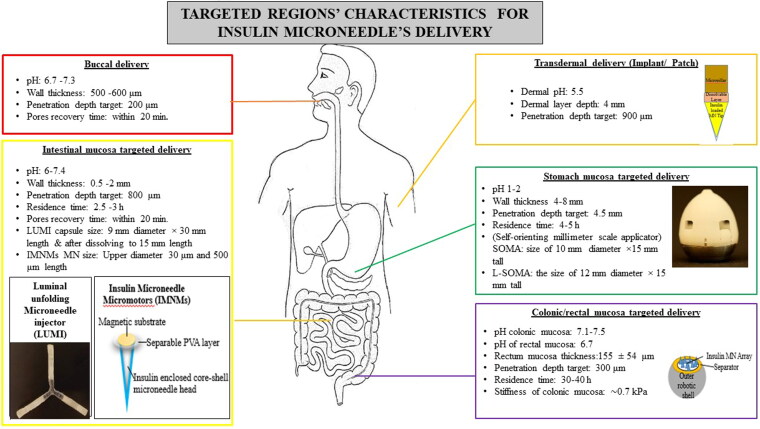
Factors taken into account for the targeted delivery of insulin microneedles. Insulin microneedle micromotors (Zhang et al., 2022): Copyright© 2022 Xiaoxuan Zhang et al. Exclusive Licensee Science and Technology Review Publishing House. Distributed under a Creative Commons Attribution License (CC BY 4.0). https://spj.science.org/doi/10.34133/2022/9797482?url_ver=Z39.882003&rfr_id=ori:rid:crossref.org&rfr_dat=cr_pub%20%200pubmed (accessed on 16 November 2023). Implantable transdermal patch: adapted and reproduced with permissions (Kim et al., 2020). Copyright (2020), Elsevier. Colonic mucosa-targeted microneedle-containing robots: adapted and reproduced with permissions (Huang et al., 2023). Copyright (2023), John Wiley & Sons, Inc.

The main limitation of the transdermal INS-MNs delivery system is the challenge and difficulty of maintaining the therapeutic level of INS. So, alternative oral MN-based INS was researched widely due to the greater acceptance and compliance with this route of delivery (Ghosh et al., 2022). Polymeric INS-MNs targeting oral mucosa, GI mucosa, and implantable sites were fabricated and evaluated for their effectiveness in delivering INS. The size morphology, mechanical strength, insertion angle, mode of insertion, and INS loading requirements differ from the transdermal MNs. These target regions are soft and delicate. Hence, the toughness and flexibility of the MN should be adjusted accordingly. These MNs for non-transdermal delivery should be capable of in-tissue biodegradation and biodistribution, capable of bending, and ease of use, with greater INS loading and should not alter the structural stability of the loaded INS. The different parameters to be considered while formulating INS-MN systems are given in Figure 4.

#### 4.3.1. Intraoral INS-MNs

MNs are applied onto the buccal mucosa and deliver the drug into the systemic circulation bypassing the intestinal and hepatic first-pass effect. Localized application at buccal mucosa yields high concentration *in situ* hence other factors like safety and long-term usage should be analyzed. In a clinical trial done to record the pain characteristics of MN insertion at buccal (1 cm behind the mouth angle, in cheek) and lip mucosa (inner lower) with stainless steel (S.S.) 750 μm long and 200 μm wide MNs, lesser pain compared to hypodermic needle and no bleeding were reported with an insertion force of 10 N (Santos et al., 2021). The advantages of this route are its neutral pH, less scarring, absence of hepatic first-pass metabolism, moistened and curved surface, and faster healing compared to other body regions. The limitation of this route is due to the continuous salivary secretion and tongue movement impeding the adhesion of the MN patch at the inserted location and hence disturbing the effective INS release. Tip-loaded polymeric MN arrays would effectively deliver INS to the buccal tissue without leakage.

In research on buccal INS-MNs, 1.0 mg of INS was delivered within 30 s in swine models. In a trial on 100 healthy volunteers, the palate was the most preferred and the sublingual region was the least preferred application site. Nearly, 95% of responders have chosen MN over injection. In the buccal mucosa and on the soft palate, greater INS release was seen. The loading efficiency was 60% and the remaining 40% was found in the base plate. The PVP-based INS MNs showed superior INS plasma levels due to their solubility and permeability than sorbitol MNs. These MNs were stable for 1 month at 25 °C. With 10 N insertion force, better penetration was observed. The pores generated by the MNs recovered within 20 min due to the fast turnover of buccal mucosa (Caffarel-Salvador et al., 2021).

Buccal mucosal delivery of INS-MN is easier as the MN system is self-administrable and there is no necessity for special activators or spring-force within the system. Patch or plunger-based MN devices can safely deposit the needle tips within the mucosa. Also, as the mucosal region could be viewed for infections or any adverse effects, it is easier to be treated than the stomach or intestinal mucosal layers.

#### 4.3.2. Gastrointestinal tract delivery (oral INS-MNs)

The main focus of oral INS-MNs is to safely administer them to the GI mucosa and to obtain better INS bioavailability comparable to injections. Drugs (epinephrine) when administered as GI injections through the layers of GI mucosa immediate response has been observed indicating systemic bioavailability. The small intestine could withstand sharp object transit of size <3 cm (Traverso et al., 2015). Also, gastroenterologists use needles on the small intestinal or colonic wall efficiently with lower complication rates (Abramson et al., 2022). Additionally, the quick healing nature of the GI mucosa from needle prick injuries by the migration of the viable epithelial cells of the GI tract to the denuded basal lamina is particularly beneficial in the case of oral INS-MN delivery (Kaffash et al., 2022).

More elaborate physical designs for INS-MNs should be designed for easier and more efficient oral INS delivery. Non-transdermal INS-MN designing aspects and the reported outcomes are summarized in Table 5. A capsule size of ‘000’ was found easier to pass through the pyloric sphincter of the stomach (Brown et al., 2019; Kaffash et al., 2022). The safest size set was 9 mm in diameter and 15 mm in length as per the FDA-approved osmotic-controlled release oral delivery system (OROS) (Abramson et al., 2019; Kaffash et al., 2022). Also, it was noticed that the onset time for hypoglycemic action with oral INS-MNs had reduced by nearly 20 min compared to S.C. INS injection (Schoellhammer et al., 2016). This indicates rapid distribution to the systemic circulation after oral INS-MN administration than the usual S.C. injection.

**Table 5.** Design characteristics and the reported findings of non-transdermal insulin microneedles.

Insulin microneedles	Polymers/excipients added	Morphological dimensions	Penetration force	Mechanical strength	MN (grind) insertion angle	Insulin loading	Findings	Reference
Intraoral MN	Biodegradable PVP	750 μm long and 200 μm wide (up to 1 mm length)	10 N	Strong enough to penetrate the palate.	–	Could load up to 2 mg of INS. 1.0 ± 0.2 mg was localized on the MN tips.	1.0 mg INS delivered within 5–30 s (swine)	Caffarel-Salvador et al. (2021)
Intragastric SOMA (self-orienting millimeter scale applicator)	Biodegradable HPMC, polyethylene oxide (PEO 200 K), and dissolvable sucrose.	Conical MN; 1.7 mm in height and 1.2 mm in diameter. Total millipost length: 7 mm	1 N	20.0 ± 0.7 MPa	10° angle	0.3–0.7 mg per pill.	Bioavailability: 10% (10–70 pM INS for 3.5 h). INS delivery coefficient: 111.1 pM/mg m^−M^. INS plasma concentration was comparable to S.C. INS injection. Zero-order release kinetics. Penetrated until muscularis mucosa above the submucosal layer of the stomach.	Abramson et al. (2019)
Intragastric l-SOMA (liquid-injectable SOMA)	MN: polyoxymethylene (non-biodegradable) Plunger: polyphenylene ether (non-biodegradable) Elastomer: Kraiburg	Needle length optimized to 4.5 mm penetration depth.	5.7 ± 2.7 N for 4.5 mm penetration depth	4.7 N	12.5 ± 2° angle	Up to 4 mg could be loaded. *In vivo* analysis done with 4 IU of INS (0.14 mg), injection flow rate: 21 μL/s	Bioavailability: 51 ± 16% for 2 h (30–81% in 2 h; *n* = 7) (80 μL delivered into the submucosal space) The set-up is made of non-biodegradable materials and should be eliminated from the body. Liquid insulin creates instability issues	Abramson et al. (2019)
Intestinal LUMI (luminal unfolding microneedle injector)	Arm with MNs: 75% 200 kDa PEO and 25 % Soluplus^®^, a biodegradable polymer. Elastomer: Mediprene Coating: Eudragit L-100/55 (20 μm thick dissolves at pH 5.5)	1 mm length and 0.4 mm base diameter.	0.20–0.28 N (human tissue) or 3 N for 30 MNs.	0.41 ± 0.06 N (13 mN per microneedle)	30° angle	0.5 mg per LUMI	LUMI *in vivo*: 40% systemic uptake. Directly applied MN intestinal patch: 80% systemic uptake. INS delivery coefficient: 81.8 pM/mg·m^−M^. Safe deployment of LUMI in the small intestinal mucosa. Delayed release due to gastric emptying time. Long-acting INS could be delivered in this way.	Abramson et al. (2019)
Intestinal IMNMs (insulin microneedle micro motors)	MN: 30% methacryloyl gelatin (GelMA) and 15% PVA: MW 89,000–98,000 Separator: PVA 30%, MW 13,000–23,000	30 μm of tip diameter and 500 μm in height.	Penetration depth is 240 μm	0.34 N	–	≈18 U of INS administered as per 6 U/kg rabbit weight.	Degradation time for MN: 40 min. Separator removal after 30 min of implantation. INS released rapidly for 2 h and then a sustained release pattern was seen for 24 h. The whole set up to be enclosed in an enteric-coated capsule to deliver the micromotor MNs intact to the intestine.	Zhang et al. (2022)
Colonic robotic micro injectors	INS-loaded chitosan gel coats	450 μm length and INS-chitosan coating thickness 5 μm.	–	0.4–0.5 MPa	–	Each microinjector holds 300 μIU of INS	INS delivery coefficient of 1036.5 pM/mg·m^−M^.	Ghosh et al. (2022)
Tumbler-inspired self-orienting MN for colon delivery	Detachable layer: PVA and PVP degradable in colonic juice (within 6 min) Degradable MN: PVA, chitosan, maleilated dextran, and polycaprolactone	–	Full penetration 0.058 N/needle for a penetration depth of 240 μm	–	@90° fall the reorientation of the device is within 0.35 s	Mechanistic study	The low center of gravity on the robotic shell makes the device self-orient vertically to its solid bottom and gets anchored to the colon mucosa for the MN to penetrate and release INS. Polymer-based dissolving MN used for long-term drug release.	Huang et al. (2023)
Powder-carrying microneedles (PCM) for transdermal implant	Micro-shells: carboxymethyl cellulose (10%)-biodegradable, biocompatible and viscous.	800.3 ± 1.4 μm height and 450.5 ± 2.1 μm diameter. Above CMC protective layer thickness 30 μm	100% micro shells penetration	PCM: 0.040 ± 0.008 N DMN: 0.033 ± 0.005 N	–	PCM carries 225.5 ± 8.6 μg of INS. DMN carries 90.4 ± 3.1 μg of INS	Increasing CMC concentration reduced the polymer microcavity dimension and increased its thickness. The amount of encapsulated cargo increased in PCM than the amount in dissolvable MN (DMN) prepared with reconstituted drug. *T*_max_: 5.40 ± 1.17 h *C*_max_: 0.96 ± 0.15 ng/mL AUC: 2.56 ± 0.94 ng·h/mL Bioavailability: 61.51%	Kim et al. (2020)

##### 4.3.2.1. Stomach targeted INS-MNs

INS-MNs pierce through the stomach wall for systemic INS distribution from a self-orienting INS delivery millipost device (SOMA). This intragastric device produced comparable bioavailability to cutaneous INS injection (Abramson et al., 2019). Intragastric INS-MNs are advantageous due to the greater thickness of the stomach wall (4–8 mm) over the intestinal wall (0.5–2 mm), faster tissue regeneration and mucous barrier’s fluidity that could quickly seal the lining defects created by the microinjection. These benefits make the stomach the better target for INS-MN delivery (Abramson et al., 2019; Kaffash et al., 2022). It was also found that the hypoglycemic onset time in the *in vivo* porcine study was more rapid for direct stomach injection than duodenum, colon and skin injections indicating immediate systemic absorption from the stomach (Traverso et al., 2015).

The limitations observed with these delivery systems include the following. (1) Low INS loading (0.3–0.7 mg per pill) and lesser bioavailability (10%). (2) Possibility of INS denaturation in the stomach fluid as the sucrose within the system should dissolve first in the stomach fluid to activate the encapsulated spring actuator to release the needle to pierce through the stomach wall. (3) 0.3 mg of INS was loaded without any polymer protective coat to overcome such degradation. (4) Some parts of the device are non-biodegradable and should be excreted from the body and (5) zero-order INS release after 3.5 h of activation.

However, recently, they have formulated l-SOMA for liquid INS. They could load up to 4 mg of different payloads. INS (0.14 mg) loaded l-SOMA demonstrated an absolute bioavailability of up to 81% over a 2 h duration for a pill during the study. This device could be prefilled with INS based on the patient’s requirement. INS gets directly released into the submucosa layers that have a greater capacity to hold liquid drug than the mucosal layer. Thus, avoiding degradation in the gastric lumen as there was no necessity for the entry of stomach fluid into the pill. The rapid release and immediate hypoglycemic onset response are beneficial for prandial INS. Comparatively, a 10° angle grind needle penetrated up to a depth of 4.5 mm, preventing deeper insertions and outer stomach perforations than a flat needle tip.

However, the main limitation of l-SOMA was due to the use of liquid INS within the device. Since INS was in its liquid state within the needle hub, protein aggregation, and purity loss were observed within 14 days. Moreover, the safety of frequent GI punctures on chronic use should be evaluated as the size of the l-SOMA needles was comparatively larger than MNs (Abramson et al., 2019). These studies on intragastric INS-MNs encourage researchers to develop new INS-based MN devices for oral delivery.

The SOMA self-orients to its piercing position based on its unique shape and in response to the gravity. However, we propose polymeric mucoadhesive coats that could be applied onto the formulated MN-containing system to fix at the adhered GI region to slowly erode and allow the MNs to pierce unidirectionally through the GI mucosa. However, still practical research on this model is not yet published.

##### 4.3.2.2. Intestine-targeted INS-MNs

MNs enclosed in a capsule inject INS into the layers of the intestine (luminal unfolding microneedle injector – LUMI). Thus overcomes the GI mucus barrier and boosts oral drug availability. Extensive surface area and vasculatures of the intestine provide better INS uptake. Abramson et al. formulated an oral INS-MN device that physically unfolds and gets inserted in the intestinal mucosa releasing INS into the intestinal layers for systemic absorption. The unfolding arms containing INS are made of biodegradable polyethylene oxide (PEO) and polyvinyl caprolactam–polyvinyl acetate–polyethylene glycol capable of biodegrading within 24 h (Abramson et al., 2019). Intestinal MNs are safer as the GI tissue regenerates to repair quickly the damage caused by the intestinal microinjection (Brown et al., 2019).

Intestinal MNs are more advantageous than other particulate oral INS delivery systems. (1) They overcome the formulation requirement on mucoadhesion, permeation enhancement, and tissue diffusion for increasing the INS uptake. (2) Thus, eliminates any additional excipients like permeation enhancer incorporation into the delivery system. (3) The force required for the intestinal MNs to enter the intestine was minimal and was between 0.2 and 0.5 N. This is due to the soft intestinal wall unlike the hard stratum corneum. (4) The length of the needle required to perforate the outermost intestinal layer was 6 mm and hence all the INS-MNs formulated using a PDMS mold of 750 μm would be safe to be inserted into the intestinal wall for INS delivery. But still, more safety analysis should be carried out. (5) This device could hold 0.3–0.6 mg of INS (Abramson et al., 2019).

The limitations reported for this delivery system are as follows. (1) Large doses of INS delivered *in situ* with rapid onset of action (0.6 mg delivered with *t*_max_ of 25 min and *C*_max_ of 46 ± 15 pM). (2) Hypoglycemia was observed in the *in vivo* studies requiring dextrose administration. (3) Long-term high-dose usage may result in toxicological mitogenic effects as INS is a cell proliferative and growth-promoting agent (Brayden, 2021). However, these issues could be solved by pre-programming the INS release *in situ.* The approach would be fabricating glucose-responsive hydrogel MNs in a biodegradable polymer matrixed with INS. The INS release from this system would be based on the bioerosion of the swollen polymer needles and dependent on the elevated amount of glucose in the body. Another approach would be by loading INS into a layer-by-layer-coated (alternative anionic INS and cationic polymer layers) biodegradable NPs, then matrixed with biodegradable polymer and made into MNs where INS release is slow and uniform based on the erosion of each coated layer.

A few factors that should be considered during the fabrication of intestinal INS-MNs are as follows. (1) Coating the MN device with the enteric coating polymer to protect from the gastric environment degradation and allow the device to reach the intestine intact. (2) In-built spring or balloon actuation (Dhalla et al., 2021) to pierce the intestinal wall should be optimized for a minimal force (0.5 N). (3) Acceptable MN length of approximately 700 μm to 1 mm for avoiding outer layer perforation. (4) Use of biodegradable/dissolvable/swellable polymer, polymeric spring, and polymeric inflating device within the capsule to maintain biosafety for long-term usage (Abramson et al., 2019).

##### 4.3.2.3. Colon/rectum targeted INS-MNs

Micromotor MNs were researched for intestinal delivery but recently, a group (Liu et al., 2022) formulated Mg^2+^-based biodegradable micromotor minitablets for oral controlled delivery of INS. This group selected the colon as the targeted site for INS delivery, paving the way for future research. These minitablet micromotors are capable of self-propelling by converting chemical energy to mechanical motion. In brief, Mg^2+^-based micromotors produce hydrogen bubbles continuously with the local body fluids and generate rapid movement driving forces (94.7563 μm s^–1^) for enhanced retention, penetration, and effective absorption of INS. Colonic INS administration is promising due to the longer residence time, thin mucosa, bypassing hepatic metabolism, and loosely arranged epithelial cells. The INS loading was found to be 5.84 ± 0.07 IU/mg. Layer-by-layer coating was performed with 0.1% chitosan (FITC labeled) and INS (rhodamine-labeled) for increasing INS loading. These Mg-INS micromotors were mixed with starch and sodium bicarbonate and were punched into mini tablets. Each 10 mg tablet contained 944 μg Mg-INS micromotors carrying 5.52 ± 0.53 IU INS. The mini tablets were coated with esterified starch for colon-targeted delivery. In *in vivo* animal studies, blood glucose was maintained at a 40% steady state for 5 h (Liu et al., 2022).

Shape-changing robotic microinjectors were origami-inspired devices that could autonomously deliver INS in GI epithelium after enteral or colon administration. The size of the microinjectors was 1.5 mm in open condition and 500 μm in closed condition. They comprise of several hinges (generates insertion force, made of Cr/Au/Ni) and tip (penetrates tissues, made of chitosan-INS) segments. These metallic materials in their elemental form are nontoxic and are widely used for biomedical applications. The injection arms were made of 450 μm length tips coated with INS-loaded chitosan gel. The thickness of the microinjector INS-loaded chitosan gel arms was 5 μm. The pre-stressed energy stored within the thin chitosan gel film releases the force when triggered by the physiological GI temperature and undergoes shape change to insert the needle tips. In the *in vitro* analysis of colonic mucosa biomimetic gelatin hydrogel (stiffness of 1 kPa), the needle pierces through 300 μm. In the *ex vivo* rat colon tissue experiment, 250 μm penetration was seen. It was also confirmed in this research that the microinjector tips penetrated only at the rat colonic mucosa and the other layers of submucosa and muscularis externa remained intact and showed the same morphology as control normal healthy rats (Ghosh et al., 2022).

The advantage of this device is instead of embedding a spring for actuation (SOMA, LUMI); the structure itself is capable of shape-changing to insert the narrow needles. The insertion pressure of the INS loaded tips was 0.4–0.5 MPa (empty unloaded tips 0.5–0.6 MPa). Because of the tip’s ultrathin thickness, the incisions created were thin and narrow and were capable of quick recovery during mucosal regeneration. Also, this article shows evidence for colonic MN delivery without any perforations in rat models of thinner colon walls than the human colon. Moreover, the number of insertion sites with SOMA was 1 and with LUMI was ≈100 (32 MN in each arm). However, the microinjectors can inject up to 600 microinjection sites. Each microinjector holds 300 μIU of INS. Microinjectors administration in rat model gave a *C*_max_ of 65.3 pM (dose administered 0.063 mg/m^2^) (Ghosh et al., 2022).

The limitations of colon-targeted INS-MN delivery would be due to the microinjectors’ transit time to reach the colon after oral intake. Also, further studies are required to analyze the long-term elemental safety of these INS delivery devices on chronic diabetic patients. Furthermore, the use of biodegradable microinjectors for INS delivery would enhance the safety and biocompatibility of these devices (Ghosh et al., 2022).

#### 4.3.3. INS-MN delivery devices coupled with external fields

A magnetic-controlled intelligent INS-MN robotic device coupled with an external magnetic field was fabricated by a group (Zhang & Shang, 2021). The device with a magnetic substrate could aid in reorienting MNs toward the small intestinal wall due to the external magnetic field and accurately pierce the needles to deliver INS. The insertion depth was 500 μm and the glucose level recovered within 2 h. Another similar research was conducted by another group with a Lego-brick-stacking-inspired setup with MN tips, magnetic substrate and a separator enclosed in an enteric capsule. On applying a magnetic field, MNs face the small intestine to pierce the tip into the intestinal tissue. The separator degrades leaving the tips within the intestinal wall for sustained INS delivery. The magnetic substrate could be safely excreted (Zhang et al., 2021).

The above mechanism was elaborated in their latest study (Zhang et al., 2022). Micromotor MNs are miniature movable MN devices that are self-driven or activated by external magnetic fields or ultrasound. INS-MN micromotors (IMNMs) were fabricated by dip-printing drawing photolithography. The biodegradable needle heads (made of 30% gelatin methacryloyl (GelMA) and 15% PVA: MW 89,000–98,000) were formed by printing the viscous gel droplets by simultaneous wiredrawing and solidifying. By repeated dipping and drawing, a single core to multilayer core–shell MN structure could be fabricated. The middle separable layer for MNs was made of dissolvable polymer (PVA 30%, MW 13,000–23,000) on the magnetic substrate basement made of polyethylene glycol diacrylate (PEGDA) and neodymium–iron–boron (NdFeB) microparticles. These IMNMs, under a magnet, were guided to take desired positions and penetrate the intestinal tissue to release INS. The penetration depth was found to be 240 μm. An initial rapid release was seen for 2 h with a subsequent plateau in diabetic rats (Zhang et al., 2022).

Schoellhammer et al. used a model device that was inserted into the rectum. INS was co-instilled as an enema in the colon with 1 min focused ultrasound resulting in an ultra-rapid hypoglycemic effect (Schoellhammer et al., 2016).

#### 4.3.4. Transdermally implantable INS-MNs

Since liquid INS is capable of degradation, INS powder carrying MN (INS-PCM) for transdermal implantation was fabricated. Liquid INS when mixed with polymer or when exposed to MN fabrication stress may be denatured losing its potency and leading to reduced efficacy. This approach provides high INS loading, sustained release, and yields long-term stable product (100% stable at −20 °C and 93.3 ± 3.8% stable for 8 weeks @25 °C). They are patchless INS-MN systems implanted transdermally using a micro-pillar. Insulin loading was found 2.5 times higher for PCM than DMNs. Also, drug saturation duration was higher for PCM (24 h) than DMN (6 h). The transepidermal water loss (TEWL) measurement indicated that it took around 9–12 h for the PCM-implanted skin to reseal. This implantable system overcomes the problem of drug loss from the base of the MNs as the whole micro-shell carrying INS power could effectively get implanted. It was observed when a dissolvable MN was inserted, there was a gap of 85.4 ± 11.7 μm between the arrays and the skin tissue. The drug located in these bases would be around 40% that gets lost (Caffarel-Salvador et al., 2021). Thus, this study proved that the implantation system was superior to the patch system for transdermal delivery (Kim et al., 2020).

## 5. Other modulated INS-MN devices: glucose-responsive insulin MN

These systems are pre-programmed for continuous smart delivery of different doses of INS dependent on the elevated BGLs for glucose hemostasis (Gu & Yu, 2021). The glucose concentration in the dermal interstitial fluid is relatable and equitable to the BGLs. Thus, the modulation of INS release based on the dermal fluid glucose level maintains normal glucose for a prolonged duration (Chen et al., 2018). These delivery devices overcome repeated finger-prick calibration to decide INS dose based on glucose levels. Glucose-sensitive substances like PBA, glucose oxidase (GOx), and concanavalin A (lignin) were researched for feedback-regulated INS delivery systems. PBA reversibly binds to glucose without producing any toxic by-products (Chen et al., 2020). The system developed was temperature-independent and had a threshold glucose-responsive quantity of 100 mg/dL (normoglycemic concentration).

In another coupling study, polymeric vesicles were integrated with the PVP/PVA MN system for fabricating dual responsive MN patches for transcutaneous INS delivery. Phenylboronic acid – a glucose-sensitive substance, GOx – interacts with glucose to generate H_2_O_2_, poly(4-(4,4,5,5-tetramethyl-1,3,2-dioxaborolan-2-yl) benzyl acrylate – a H_2_O_2_ sensitive substance, were added as selective functional groups in the polymeric vesicles. Polymeric vesicles were made of amphiphilic triblock copolymer, PEG-*b*-poly-(3-acrylamido PBA)-*b*-poly(4-(4,4,5,5-tetramethyl-1,3,2-dioxaborolan-2-yl)benzylacrylate) to load the hydrophilic INS. It was found that when the glucose level was higher, GOx interacts with glucose to produce peroxides that sensitize the H_2_O_2_-sensitive substance to undergo disassembly of the vesicles. In high glucose levels, faster release of INS from the system occurred due to PBA group sensitivity. The mechanical strength of the PVP/PVA MN with vesicles was 0.168 N. The INS release from the system was dependent on the glucose concentration and H_2_O_2_ level but independent of the INS dose (Tong et al., 2018). This on-and-off INS release mechanism with MNs could be researched on oral and GI MN devices too in the future.

## 6. Limitations

Insulin is a hormone that is considered proliferative in some cases. There were no long-term studies on the cytotoxicity and biocompatibility of the INS MNs delivered through different routes. The dermal skin is packed with antigen-response cells that may provoke immune responses when a foreign drug is administered intradermally. Also, repeated MN insertion at the same site may trigger immunological reactions. So, long-term immunological safety should be analyzed. Sterility, endotoxin limit, particulate matter, and water activity should be checked for MNs to avoid clinical infections in the patients (Dul et al., 2023).

Polymer selection for MNs should be done based on the nature of biodegradation and the required MN mechanical strength. The amount of residual polymer left behind underneath the skin after drug absorption should be evaluated. Insulin should be taken by a diabetic patient for a long period and hence even a minute polymer deposition could trigger an immunological reaction, and accumulation in liver or body tissues over time. The kinetics of polymer clearance, removal method of deposited polymer, and long-time safety profile should be researched in-depth for various routes of MN delivery.

The drug payload in the MN array is minimal due to the smaller mold capacity. Hence, the frequency of MN administration should be evaluated carefully for diabetic patients. The *in vitro in vivo* correlation (IVIVC) studies should be carried out to compare the S.C. and MN-delivered INS dose with the pharmacokinetic and pharmacodynamic responses. The amount of drug loaded into the needle should be correlated proportionally with the plasma concentration or the cumulative release profile (Wang et al., 2019). Moreover, coating INS with polymeric layers or core–shell NPs of INS or glucose-responsive on-and-off system provides longer release kinetics within the body as the short-lived INS is getting released based on the requirement or polymer degradation.

Most of the non-transdermal INS-MNs researched were found to produce a rapid immediate hypoglycemic response within 30 s (Caffarel-Salvador et al., 2021) or 30 min (Abramson et al., 2019). Few of them have reported controlled release or sustained release from their systems, which is essential to maintain glycemic hemostasis. Also, the complex nature of the 3D printed devices and their size optimization to pass through the entire GI tract without obstruction were the issues to be addressed. In a study, it took around 7–56 days for the device to get expelled out, hence human clinical trials on safety and efficacy should be assessed too. Hence, patient-related factors like gastric emptying time (longer duration for diabetic patients) should be considered during the device fabrication. Thus, the use of biodegradable parts for the whole device design would enhance the safety and efficacy of the product. The S.S. spring forces would be strong and may perforate the GI barriers (Ghosh et al., 2022). So, dissolvable polymeric springs made of safe and biodegradable ‘shape memory polymers (SMPs)’ could be used alternatively and that too requires elaborate testing on insertion force and penetration depth analysis.

## 7. Future perspective

Novel fabrication techniques should be elucidated for maximum drug loading and coating. More innovations or approaches should be carried out on the dose adjustability, as cutting the MN patch by the patient for application may lead to under or over-dosing risks. Also, an approximate guideline on the time duration that the patch should be applied for the complete detachment of needles from the backing layer should be evaluated. Moreover, for dissolving needles, after insertion, a snap-sound or audible endpoint system should be developed for patient ease to check on the complete insertion.

Insertion capability and dissolution or biodegradation mechanism of the needles beneath the skin should be extensively studied. In a study, approximately 40% of the MN remained in the base plate after the removal from the skin (Zhang et al., 2021). So, it is important to load INS first and then the polymer alone above to avoid INS waste. Thus, there is a research gap in formulating fully insertable MNs. Based on the micro-pillars and implantable MNs discussed in this article, newer approaches could be studied to address this issue. Polymer flexibility is crucial to ensure better MN insertion without compromising its hardness at various insertion sites (Yuzhakov, 2010).

Even after the use of MNs, the patch may or may not contain drug-loaded MNs. An appropriate packaging system with a disposal holder for placing the after-use MNs should be made. Henceforth, we can avoid the potential sharp risks and the contamination risk of blood and interstitial fluid. Also, there should be some technique to avoid the reuse of MNs patch, again and again, to prevent blood-borne pathogenic infections in patients. A patient-friendly slide-and-seal microneedle array patch (MAP) box resembling a conventional pill box was designed to improve patient adherence and enhance MN protection (Anjani et al., 2023).

For any oral INS to be approved, the safety of the drug delivery system is the main concern. Evaluating the cellular membrane porosity after MN insertion, its recovery duration, biological factors responsible for dose dumping, incomplete dose release, and dose variability should be evaluated. Innovative polymeric dissolvable MN composites can be fabricated into bioinspired designs for payload delivery (Liu et al., 2023). However, factors like polymer selection, nature-inspired design, and mechanical and chemical aspects should be evaluated thoroughly to construct an effective INS-MN system.

## 8. Conclusions

Replacing S.C. injections with an oral INS delivery system is essential as the diabetic population and diabetes complications are increasing every year. People prefer oral therapy rather than injections as evidenced by the oral use of semaglutide (Rybelsus^®^) and octreotide (Mycapssa^®^). When compared with FDA-approved OROS osmotic tablets, the physical INS-MN devices constructed by the researchers are of similar morphological sizes. Additionally, the insertion force requirement from polymer INS-MNs for soft and moist tissues of the oral and GI tract would be comparatively lesser than the transdermal MNs. Hence, more research focusing on these routes with MN delivery systems should be done on various macromolecules. Successful INS delivery through non-transdermal MN delivery will pave the way for living cargoes like therapeutic stem cells, probiotics, or genetic materials to be targeted through smart ingestible devices.

## Data Availability

Data sharing is not applicable to this article as no new data were created or analyzed in this review.
